# P-485. Healthcare-associated Respiratory Syncytial Virus (RSV) Infection During Prolonged Birth Hospitalization: Is There a Role for Nirsevimab?

**DOI:** 10.1093/ofid/ofaf695.700

**Published:** 2026-01-11

**Authors:** Pablo J Sanchez, Sarah King, Helena Brenes-Chacon, Matthew Washam, Samiksha Tarun, Stacie Rhoades, Cristina Tomatis Souverbielle, Asuncion Mejias

**Affiliations:** Nationwide Children's Hospital, Columbus, OH; Nationwide Children's Hospital, Columbus, OH; St. Jude Children's Research Hospital, Germantown, TN; Nationwide Children’s Hospital, Columbus, Ohio; Nationwide Children's Hospital/OSU, Columbus, Ohio; Nationwide Children's Hospital, Columbus, OH; Nationwide Children's Hospital, Columbus, OH; St Jude Children's Research Hospital, Memphis, TN

## Abstract

**Background:**

On 8/3/2023, the CDC/Advisory Committee on Immunization Practices recommended nirsevimab, a long-acting monoclonal antibody, for prevention of RSV-associated lower respiratory tract infection in all infants aged < 8 months and some high-risk young children aged 8-19 months. At Nationwide Children’s Hospital (NCH; 673 beds) and its 7 affiliated NICUs ( > 3,000 admissions/year) in Columbus, OH, infants with prolonged birth hospitalization related to prematurity or other causes receive nirsevimab shortly before hospital discharge. Earlier use of nirsevimab for prevention of healthcare-associated (HA) RSV disease was not recommended. However, HA-RSV disease is associated with substantial morbidity and occasional mortality, mainly in children with underlying medical conditions. Our objective was to determine the magnitude and review the cases of HA-RSV infections at NCH and its NICUs in order to inform guidance on timing of nirsevimab administration.

**Figure:**
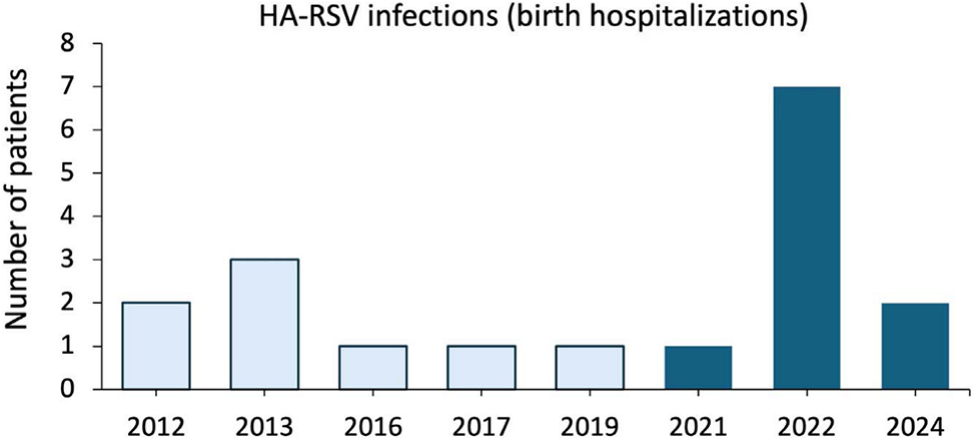
Healthcare-associated RSV infections during the birth hospitalization of 18 infants and young children <2 years of age

**Methods:**

Retrospective analysis of prospectively obtained surveillance data of all HA-RSV infections that occurred during infants’ birth hospitalization. RSV PCR testing in infants who developed respiratory symptoms was at the discretion of the attending physician.

**Results:**

From 2012 to 2024, there were 76 cases of HA-RSV infections in infants and children < 2 years of age. Of the 76 cases, 18 (24%) occurred among infants during the birth hospitalization for a median of 1-2 cases/year with a range of 1 to 7 infants per year (Fig 1). Median (IQR) chronologic age at the time of the HA-RSV infection was 1.7 (0.8-3.5) months. 72% (13/18) of infants (gestational age, 22 to 40 wks; birth weight, 550 to 3590 g) were in the NICU (10, preterm; 4, BPD; 3, genetic disorder) while 2 (gestational age, 39 and 37 wks; birth weight, 3145 g and 3200 g) were in the Cardiothoracic-ICU (1, transposition of the great arteries, s/p SWITCH; 1, cardiomyopathy, pulmonary hypertension, s/p ECMO). None of the infants had received RSV immunoprophylaxis and none died.

**Conclusion:**

HA-RSV disease was infrequent among infants during the birth hospitalization. Use of nirsevimab in addition to traditional infection prevention and control strategies would have resulted in immunization of many infants in order to prevent few HA-RSV cases.

**Disclosures:**

Cristina Tomatis Souverbielle, MD, Merck inc: Research support, isp Asuncion Mejias, MD, PhD, MsCS, Enanta: Advisor/Consultant|Merck: Grant/Research Support|Moderna: Advisor/Consultant|Pfizer: Advisor/Consultant|Sanofi-Pasteur: Advisor/Consultant

